# Editorial for Special Issue “Molecular Insights into Food-Derived Natural Products and Their Biological Activities”

**DOI:** 10.3390/cimb47070480

**Published:** 2025-06-21

**Authors:** Yanfang Li

**Affiliations:** 1 Methods and Application of Food Composition Laboratory, Beltsville Human Nutrition Research Center, Agricultural Research Service, U.S. Department of Agriculture, Beltsville, MD 20705, USA; yanfang.li@usda.gov; 2 Department of Chemistry and Biochemistry, Ohio University, Athens, OH 45701, USA

Food-derived natural products offer more than just essential nutrients like vitamins, calcium, iron, zinc, selenium, and so on. They also contain a rich array of phytochemicals that including flavonoids, phenolic acids, triterpenoids, polysaccharides, fatty acids, steroids, proteins, and peptides, which show potential in preventing and treating various chronic diseases [1]. Their health-promoting benefits are extensive, encompassing anti-inflammatory, antioxidant, anti-stress, antimicrobial, antifungal, antibacterial, anti-aging, and anti-cancer properties, as well as cardiovascular benefits and glucose, lipid metabolism, gut microbiota, and immune system regulation [1]. LC-MS-based non-targeted and targeted metabolomics are now the most important and advanced technologies for exploring the complex chemical profiles of these food-derived natural products, which is essential to understanding their protective mechanisms at the molecular level [2,3]. With an increasing preference for natural foods over synthetic ingredients, greater efforts are being made to explore their functional components and health benefits, along with their molecular mechanisms, to advance the development of new functional products. Therefore, this Special Issue brings together ten contributions, including five research articles [4,5,6,7,8] and five reviews [9,10,11,12,13], that highlight the diverse biological functions, diverse therapeutic applications, and the related mechanistic insights of several food-derived natural products.

The issue opens with a study conducted by Park et al., focusing on investigating the effects of *Eruca sativa*-derived flavonols on skin barrier function, employing quantitative analysis and molecular docking simulations to elucidate their protection mechanisms [4]. Similarly, Peno-Mazzarino et al. investigated a botanical mixture containing extracts of *Lippia citriodora*, *Olea europaea*, *Rosmarinus officinalis*, and *Sophora japonica* for its protective effects against environmental pollution, demonstrating its potential benefits for both cutaneous and cardiopulmonary systems [7]. Further expanding on natural compounds, Shih et al. investigated the synergistic effects of epigallocatechin-3-gallate (EGCG), a prominent green tea polyphenol, in enhancing arecoline-induced cytotoxicity and promoting apoptosis, thereby offering new perspectives for cancer therapeutics [5]. Tang et al. identified the secondary metabolites from *Camellia fascicularis* leaves with antioxidant and antimicrobial properties, reinforcing the importance of phytochemicals in health applications [6]. Go et al. delved into the functional activities and mechanisms of *Aronia melanocarpa*, revealing its contributions in preventing various diseases to overall well-being [9]. Tzimas et al. also highlighted various plant-derived compounds as promising tools for dental caries prevention, underscoring their antimicrobial and antibiofilm protection effects against oral pathogens [10].

In addition, Shea et al. reviewed legume-derived bioactive compounds, such as peptides, protein subunits, antinutritional factors, saponins, and isoflavones, focusing on their impact on human and animal health, particularly in nutrition and disease prevention [11]. Concurrently, Lee et al. investigated the role of soy isoflavones in inducing prostate cancer cell apoptosis via the inhibition of STAT3, ERK, and AKT pathways, providing valuable insights into plant-based cancer treatments [8]. In another study, Tian et al. reviewed the effects of pterostilbene in cardiovascular disease management, emphasizing its potential therapeutic applications [12]. Apalowo et al. reviewed microbial-derived bioactive compounds in alleviating inflammation in obesity, further contributing to the growing field of gut microbiome research and metabolic health [13].

Collectively, these studies significantly advance our understanding of bioactive compounds from several valuable natural food resources and their broad applications in human health. From skin health to metabolic disorders, cardiovascular protection, oral care, and cancer prevention, this Special Issue effectively demonstrates the far-reaching potential of botanical-based bioactive compounds. Furthermore, these contributions offer a multi-dimensional understanding of how food-derived compounds can influence human health at the molecular level.

We hope this Special Issue serves as a valuable resource for researchers and inspires further studies in exploring the protection potential of natural botanical-based compounds in molecular nutrition and expanding their applications in health and disease management. Finally, we extend our sincere gratitude to all authors and peer reviewers for their valuable contributions to this Special Issue and welcome more submissions for its second edition.
